# Real‐world use and outcomes of dolutegravir‐containing antiretroviral therapy in HIV and tuberculosis co‐infection: a site survey and cohort study in sub‐Saharan Africa

**DOI:** 10.1002/jia2.25961

**Published:** 2022-07-18

**Authors:** Matthew L. Romo, Ellen Brazier, Dominique Mahambou‐Nsondé, Reneé De Waal, Christine Sekaggya‐Wiltshire, Cleophas Chimbetete, Winnie R. Muyindike, Gad Murenzi, Cordelia Kunzekwenyika, Thierry Tiendrebeogo, Josephine A. Muhairwe, Patricia Lelo, Anastase Dzudie, Christelle Twizere, Idiovino Rafael, Oliver C. Ezechi, Lameck Diero, Marcel Yotebieng, Lukas Fenner, Kara K. Wools‐Kaloustian, N. Sarita Shah, Denis Nash, International epidemiology Databases to Evaluate AIDS (IeDEA)

**Affiliations:** ^1^ Department of Epidemiology and Biostatistics & Institute for Implementation Science in Population Health CUNY Graduate School of Public Health and Health Policy City University of New York New York New York USA; ^2^ Centre de traitement ambulatoire de Brazzaville enceinte CHU de Brazzaville Brazzaville Congo; ^3^ Centre for Infectious Disease Epidemiology and Research School of Public Health and Family Medicine University of Cape Town Cape Town South Africa; ^4^ Infectious Diseases Institute Makerere University Kampala Uganda; ^5^ Newlands Clinic Harare Zimbabwe; ^6^ Department of Internal Medicine Faculty of Medicine Mbarara University of Science and Technology Mbarara Uganda; ^7^ Research for Development (RD Rwanda) and Rwanda Military Hospital Kigali Rwanda; ^8^ SolidarMed Masvingo Zimbabwe; ^9^ University of Bordeaux Inserm French National Research Institute for Sustainable Development (IRD) Bordeaux Population Health Research Center Bordeaux France; ^10^ SolidarMed Maseru Lesotho; ^11^ Kalembelembe Pediatric Hospital Kinshasa Democratic Republic of the Congo; ^12^ Clinical Research Education Networking and Consultancy Yaoundé Cameroon; ^13^ Centre National de Référence en matière de VIH/SIDA (CNR) Bujumbura Burundi; ^14^ SolidarMed Ancuabe Mozambique; ^15^ Clinical Sciences Department Nigerian Institute of Medical Research Lagos Nigeria; ^16^ School of Medicine College of Health Sciences Moi University Eldoret Kenya; ^17^ Department of Medicine Albert Einstein College of Medicine Bronx New York USA; ^18^ Institute of Social and Preventive Medicine University of Bern Bern Switzerland; ^19^ Department of Medicine Indiana University School of Medicine Indianapolis Indiana USA; ^20^ Division of Infectious Diseases Emory University School of Medicine & Emory University Rollins School of Public Health Atlanta Georgia USA

**Keywords:** antiretroviral agents, HIV integrase inhibitors, antitubercular agents, rifampin, drug interactions, observational study

## Abstract

**Introduction:**

Dolutegravir is being scaled up globally as part of antiretroviral therapy (ART), but for people with HIV and tuberculosis co‐infection, its use is complicated by a drug–drug interaction with rifampicin requiring an additional daily dose of dolutegravir. This represents a disadvantage over efavirenz, which does not have a major drug–drug interaction with rifampicin. We sought to describe HIV clinic practices for prescribing concomitant dolutegravir and rifampicin, and characterize virologic outcomes among patients with tuberculosis co‐infection receiving dolutegravir or efavirenz.

**Methods:**

Within the four sub‐Saharan Africa regions of the International epidemiology Databases to Evaluate AIDS consortium, we conducted a site survey (2021) and a cohort study (2015–2021). The cohort study used routine clinical data and included patients newly initiating or already receiving dolutegravir or efavirenz at the time of tuberculosis diagnosis. Patients were followed from tuberculosis diagnosis until viral suppression (<1000 copies/ml), a competing event (switching ART regimen; loss to program/death) or administrative censoring at 12 months.

**Results:**

In the survey, 86 of 90 (96%) HIV clinics in 18 countries reported prescribing dolutegravir to patients who were receiving rifampicin as part of tuberculosis treatment, with 77 (90%) reporting that they use twice‐daily dosing of dolutegravir, of which 74 (96%) reported having 50 mg tablets available to accommodate twice‐daily dosing. The cohort study included 3563 patients in 11 countries, with 67% newly or recently initiating ART. Among patients receiving dolutegravir (*n* = 465), the cumulative incidence of viral suppression was 58.9% (95% confidence interval [CI]: 54.3–63.3%), switching ART regimen was 4.1% (95% CI: 2.6–6.2%) and loss to program/death was 23.4% (95% CI: 19.7–27.4%). Patients receiving dolutegravir had improved viral suppression compared with patients receiving efavirenz who had a tuberculosis diagnosis before site dolutegravir availability (adjusted subdistribution hazard ratio [aSHR]: 1.47, 95% CI: 1.28–1.68) and after site dolutegravir availability (aSHR 1.28, 95% CI: 1.08–1.51).

**Conclusions:**

At a programmatic level, dolutegravir was being widely prescribed in sub‐Saharan Africa for people with HIV and tuberculosis co‐infection with a dose adjustment for the drug–drug interaction with rifampicin. Despite this more complex regimen, our cohort study revealed that dolutegravir did not negatively impact viral suppression.

## 1 INTRODUCTION

Dolutegravir, an integrase strand transfer inhibitor (InSTI), is recommended as part of preferred antiretroviral therapy (ART) regimens for people with HIV (PWH) [1, 2]. Globally, HIV treatment programs are transitioning from non‐nucleoside reverse transcriptase inhibitors (NNRTIs) [3, 4], such as efavirenz, to dolutegravir because of its superior efficacy and tolerability, and high genetic barrier to HIV drug resistance [5, 6]. The recommendation for dolutegravir is also inclusive of patients with tuberculosis co‐infection [1, 2]. Tuberculosis is a leading cause of morbidity and mortality among PWH globally [7] and affected approximately 815,000 PWH in 2019, among whom more than 70% were in sub‐Saharan Africa [8].

For patients with tuberculosis co‐infection, the use of dolutegravir is complicated by a drug–drug interaction with rifampicin [9], which is part of the standard 6‐month treatment regimen for drug‐susceptible tuberculosis [10]. Rifampicin induces the expression of UDP‐glucuronosyltransferase 1A1 (UGT1A1) and cytochrome P450 3A4, resulting in increased metabolism of dolutegravir and subsequently reduced plasma concentrations [9, 11, 12]. The reduced dolutegravir plasma concentrations can be overcome by increasing dolutegravir dosing from 50 mg once daily to 50 mg twice daily, maintained for 2 weeks after the last dose of rifampicin [9, 13, 14]. The more complicated management of this interaction is a disadvantage over efavirenz, which has no major drug–drug interaction with rifampicin [15]. In the International Study of Patients with HIV on Rifampicin ING (INSPIRING) trial, twice‐daily dolutegravir was safe and well‐tolerated among PWH receiving rifampicin‐containing tuberculosis treatment [16]. Except for this trial, the recommendation for dolutegravir among patients with tuberculosis co‐infection is largely extrapolated from trials among PWH without tuberculosis [1, 2, 6].

We sought to (1) describe HIV clinic practices for prescribing concomitant dolutegravir and rifampicin and (2) characterize virologic outcomes among patients with tuberculosis co‐infection receiving dolutegravir and make comparisons with patients receiving efavirenz.

## 2 METHODS

The data for this study came from the International epidemiology Databases to Evaluate AIDS (IeDEA) consortium, which collects routine clinical data from HIV care and treatment sites in 44 countries across seven geographical regions. This study, which focused on the four sub‐Saharan Africa regional cohorts (Central, East, Southern and West; Appendix S1: members of the contributing IeDEA regions), consisted of a cross‐sectional site survey of HIV clinics and a patient‐level cohort study. Institutional review boards in each country and regional data management centre provided ethical oversight and approved the use of de‐identified patient data. Consent requirements for study participation were deferred to local institutional review boards.

### 2.1 Site survey

We developed a survey about site practices for prescribing concomitant dolutegravir and rifampicin (Appendix S2) and successfully piloted it at one site. The survey asked about routine practices in the clinic and the respondent was instructed to consult other staff as needed to answer the questions. From August through October 2021, the survey was completed in English or French by clinical or administrative staff via Research Electronic Data Capture (REDCap) [17, 18]. We included 109 sites that reported prescribing dolutegravir in a multiregional site survey conducted in 2020 at 137 IeDEA sites in sub‐Saharan Africa [19]. Sites were excluded from the analysis if they did not provide ART to patients with tuberculosis disease (i.e. referring them to other sites) or stated that dolutegravir was not being prescribed at their site.

### 2.2 Cohort study

The cohort study used patient‐level data collected at sites during routine clinical encounters for HIV care, through point of care data entry directly into an electronic health record or on paper forms later extracted into local databases. Data were de‐identified before being transmitted to regional data management centres for processing and harmonization using an established data exchange standard [20].

### 2.3 Inclusion and exclusion criteria

We included sites that had begun to prescribe dolutegravir and were in countries with national guidelines that recommended dolutegravir as part of the preferred first‐line ART regimen, including among patients with tuberculosis co‐infection. We included patients who were ≥15 years old at HIV care enrolment and had a diagnosis of active tuberculosis from 24 months before their site began to use dolutegravir until 12 months before their site's database closure date. A diagnosis of tuberculosis was defined as documentation of a clinician's diagnosis or a proxy diagnosis based on documentation of initiating the standard regimen of isoniazid, rifampicin, pyrazinamide and ethambutol (HRZE) for drug‐susceptible tuberculosis [21]. Additional inclusion criteria were for patients already on ART to be receiving efavirenz or dolutegravir at the time of tuberculosis diagnosis and for ART‐naïve patients to initiate ART with efavirenz or dolutegravir within 2 months after tuberculosis diagnosis.

To reduce potential misclassification of ART regimen at tuberculosis diagnosis, we excluded patients with conflicting ART regimen start dates (e.g. same start date for efavirenz and dolutegravir), likely resulting from data entry errors, as well as patients with a documented change in ART regimen (e.g. from dolutegravir to efavirenz) within 1 month after their tuberculosis diagnosis. ART regimens were defined as first‐ and second‐line regimens recommended by the World Health Organization (WHO) [1, 2], consisting of an NNRTI, InSTI or protease inhibitor (PI) and dual‐nucleoside/nucleotide reverse transcriptase inhibitor (NRTI) backbone.

### 2.4 Measures

**Figure 1:**
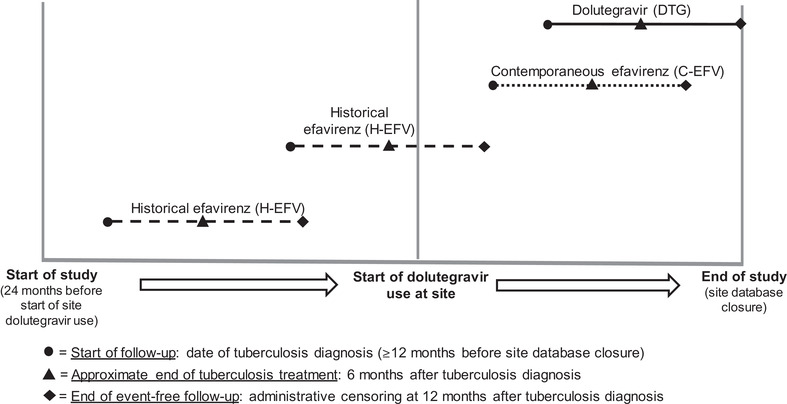
Exposure group assignment by site start of dolutegravir use in the patient‐level cohort study. The horizontal axis represents time, beginning at 24 months before the start of dolutegravir use at the patient's site and ending at the site date of database closure. Each individual line represents hypothetical event‐free 12‐month follow‐up for the three exposure groups: historical efavirenz (H‐EFV; dashed line), contemporaneous efavirenz (C‐EFV; dotted line) and dolutegravir (DTG; solid line). Patients who were receiving or initiating efavirenz at the time of a tuberculosis diagnosis were divided into two control groups: C‐EFV, with tuberculosis diagnosis after the patient's site began using dolutegravir and H‐EFV with tuberculosis diagnosis before the patient's site began using dolutegravir.

Our exposure was receiving either a dolutegravir‐ or efavirenz‐containing regimen at the time of tuberculosis diagnosis or initiating ART with such a regimen within 2 months after tuberculosis diagnosis. Patients receiving efavirenz comprised the control groups for comparison with dolutegravir and were stratified by timing of tuberculosis diagnosis, either before the introduction of dolutegravir at their site (historical efavirenz, “H‐EFV”) or after the introduction of dolutegravir at their site (contemporaneous efavirenz, “C‐EFV”) (Figure 1). The inclusion of the H‐EFV group allowed for a comparison when dolutegravir was not yet prescribed, potentially minimizing selection bias versus the comparison with the C‐EFV group.

Our outcome was viral suppression, which was defined as the earliest documentation of a viral load (VL) measurement <1000 copies/ml during follow‐up. VL was based on documentation of a quantitative HIV RNA test using local site protocols and procedural standards. A threshold of 1000 copies/ml was chosen to reflect variation across sites in specimens used and assay lower limits of detection. Based on site testing capacity [22], we assumed that most patients would have VL ascertained during follow‐up, and based on WHO guidelines [23], most would have one measurement, with multiple measurements among those with an unsuppressed VL.

Competing events were switching to a new ART regimen and loss to program or death, as these events could either modify the risk or prevent the occurrence of the viral suppression outcome. Switching to a new ART regimen was based on medication records, indicating the discontinuation of efavirenz or dolutegravir, that is switch from efavirenz to dolutegravir, a PI or another NNRTI, or switch from dolutegravir to an NNRTI or a PI. Reasons for regimen switches were not documented but were likely related to issues specific to the patient (e.g. tolerability) or program (e.g. antiretroviral availability). Loss to program included loss to follow‐up (i.e. no record for ≥6 months immediately before administrative censoring at 12 months after tuberculosis diagnosis), known facility transfer or other documented reason for leaving care, with the last recorded date of contact used to determine the timing of this outcome. Determination of death was based on a site's usual practices for ascertainment.

Baseline variables were age group, sex, underweight (i.e. body mass index <18.5; based on measured height and weight closest to the date of tuberculosis diagnosis), documentation of a prior AIDS diagnosis (i.e. CD4 count <200 cells/mm^3^ or CD4^+^ percentage <14, or WHO Clinical Stage 4 event), initial NRTI backbone, time of ART initiation, CD4 count closest to date of tuberculosis diagnosis (within 12 months before or 2 months after), most recent VL in the previous 12 months among those on ART for >6 months at tuberculosis diagnosis and site of tuberculosis disease (i.e. pulmonary only, extrapulmonary or unknown due to a proxy diagnosis). Documentation of an HRZE regimen included both patients with a proxy diagnosis and patients who initiated HRZE within 1 month of a documented tuberculosis diagnosis. As the collection and completeness of data on tuberculosis medications varied by site, we limited this variable to sites with ≥1 patient documented to receive HRZE.

### 2.5 Statistical analysis

For the site survey, we computed frequencies and proportions of all variables. For the cohort study, we computed descriptive statistics for patient characteristics and variables pertaining to data availability, overall and stratified by exposure group, and used Pearson's chi‐squared tests (categorical variables) and Mann–Whitney U tests (numeric variables) to compare DTG with C‐EFV and H‐EFV.

Patients were followed from their date of tuberculosis diagnosis until documentation of viral suppression, a competing event (i.e. switching ART regimen, loss to program or death) or administrative censoring at 12 months after tuberculosis diagnosis. If the viral suppression outcome was documented on the same day as a competing event, the patient was considered to have the former outcome.

We computed crude cumulative incidence proportions with 95% confidence intervals (CIs) for viral suppression and competing events using the Aalen–Johansen estimator [24]. We used multivariable Fine–Gray subdistribution hazards regression models [25] to estimate hazard ratios for viral suppression, comparing DTG with C‐EFV and H‐EFV. For each comparison, covariables in the model were patient characteristics that were significantly different between groups (*p*<0.05) in bivariate analyses, except for CD4 count, which was expected to be influenced by the decreasing use of CD4 testing over time [26]. In our assessment of viral suppression, we also stratified analyses by time of ART initiation, HRZE regimen documentation and site of tuberculosis disease. Cumulative incidence and regression analyses were conducted among the entire sample and among patients with VL ascertained in the 12 months after tuberculosis diagnosis. We conducted three sensitivity analyses: redefining viral suppression as <400 copies/ml; stratifying by the number of VL tests in the 12 months after tuberculosis diagnosis and by recent baseline VL among those initiating ART >6 months before tuberculosis diagnosis.

## 3 RESULTS

### 3.1 Site survey

Of 109 potentially eligible sites, 97 (89%) responded to the survey, of which seven were excluded because they referred patients with tuberculosis co‐infection to other sites for management (*n* = 5) or did not use dolutegravir at their site (*n* = 2). The 90 sites that were included were in 18 countries, with 61 (69%) in an urban location and 47 (55%) with a primary level of care (Table S1).

Overall, 86 (96%) sites reported prescribing dolutegravir to PWH who were receiving rifampicin‐containing tuberculosis treatment. Among these sites, routine practices regarding dolutegravir dosing varied: 69 (80%) reported that they start all patients on a second daily dose of dolutegravir, nine (10%) reported that all patients receive a once‐daily dolutegravir regimen and eight (9%) reported that only some patients are started on a second daily dose. For the latter eight sites providing twice‐daily dosing only to some patients, patient groups that do not routinely receive twice‐daily dolutegravir include those with a history of adverse events or tolerability issues related to ART (*n* = 5), those with low body weight (*n* = 4) and those at risk for suboptimal ART adherence (*n* = 1). Among the 77 sites that reported using twice‐daily dosing, 74 (96%) reported having dolutegravir 50 mg tablets available on the day of the survey.

Overall, four (4%) sites reported never prescribing dolutegravir to PWH who were receiving rifampicin‐containing tuberculosis treatment. Of these sites, two reported routinely starting patients not yet on ART at the time of tuberculosis diagnosis on efavirenz‐containing regimens. For patients already on dolutegravir at the time of tuberculosis diagnosis, the same two sites reported switching these patients to an efavirenz‐containing regimen while receiving rifampicin. Practices at the other two sites were not ascertained. None of the four sites reported using other rifamycins.

### 3.2 Cohort study

**Figure 2:**
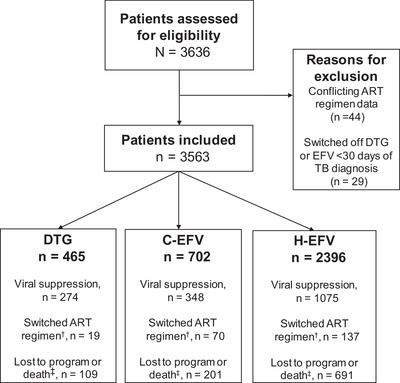
Assessment for eligibility and reasons for exclusion, exposure group disposition and outcomes. Abbreviations: ART, antiretroviral therapy; C‐EFV, contemporaneous efavirenz; DTG, dolutegravir; EFV, efavirenz; H‐EFV, historical efavirenz; TB, tuberculosis. ^†^Details on regimen switches for the DTG group: *n* = 13 switched to efavirenz and *n* = 6 switched to a protease inhibitor (PI)‐containing regimen (atazanavir/ritonavir or lopinavir/ritonavir); details on regimen switches for the C‐EFV group: *n* = 46 switched to dolutegravir, *n* = 20 switched to a PI‐containing regimen and *n* = 4 switched to nevirapine; details on regimen switches for the H‐EFV group: *n* = 41 switched to dolutegravir, *n* = 63 switched to a PI‐containing regimen and *n* = 33 switched to nevirapine. ^‡^Specific outcomes related to loss to program/death for the DTG group: *n* = 47 were lost to follow‐up, *n* = 31 had a known reason for leaving care, *n* = 31 had death documented; specific outcomes related to loss to program/death for the C‐EFV group: *n* = 96 were lost to follow‐up, *n* = 41 had a known reason for leaving care, *n* = 64 had death documented; specific outcomes related to loss to program/death for the H‐EFV group: *n* = 400 were lost to follow‐up, *n* = 104 had a known reason for leaving care, *n* = 187 had death documented.

Overall, 3636 patients met inclusion criteria of whom 73 were excluded, 44 for conflicting ART regimen start dates and 29 due to switching ART regimen within 1 month of tuberculosis diagnosis (Figure 2). This resulted in 3563 patients from 65 sites in 11 countries who had tuberculosis diagnosed from March 2015 through October 2020, with site database closure ranging from October 2019 through November 2021. Patients were in Kenya (1622 [46%]), Uganda (639 [18%]), Cameroon (350 [10%]), Rwanda (282 [8%]), Tanzania (206 [6%]), Zimbabwe (188 [5%]), Republic of the Congo (75 [2%]), Lesotho (65 [2%]), Burundi (61 [2%]), Mozambique (38 [1%]) and the Democratic Republic of the Congo (37 [1%]).

Most patients (65%) were between 30 and 49 years old, 53% were male, 43% were underweight, 44% had a prior AIDS diagnosis and 67% initiated ART at the time of tuberculosis diagnosis or within 6 months prior (Table 1). Among those with a CD4 test (39%), the median count was 135 cells/mm^3^. Among patients on ART >6 months, 62% had a recent VL test, of whom 24% had a VL ≥1000 copies/ml. Most patients (61%) had a diagnosis of pulmonary only tuberculosis, 8% had a diagnosis of extrapulmonary tuberculosis and 32% had a proxy diagnosis. Among patients at sites reporting tuberculosis medications, 72% had documentation of HRZE.

**Table 1:** Characteristics of patients at tuberculosis diagnosis and data availability, overall and by dolutegravir and efavirenz exposure groups

		Exposure group			*p*‐Value for comparison^a^	
Variable	Total	DTG	C‐EFV	H‐EFV	DTG versus C‐EFV	DTG versus H‐EFV
Total, *n* (row %)	3563 (100)	465 (13.1)	702 (19.7)	2396 (67.3)	NA	NA
*Patient characteristics*						
Age group, *n* (column %)						
15–30 years	644 (18.1)	55 (11.8)	155 (22.1)	434 (18.1)	<0.001	<0.001
30–39 years	1277 (35.8)	140 (30.1)	254 (36.2)	883 (36.9)		
40–49 years	1022 (28.7)	150 (32.3)	181 (25.8)	691 (28.8)		
≥50 years	620 (17.4)	120 (25.8)	112 (16.0)	388 (16.2)		
Sex, *n* (column %)						
Male	1882 (52.8)	313 (67.3)	320 (45.6)	1249 (52.1)	<0.001	<0.001
Female	1681 (47.2)	152 (32.7)	382 (54.4)	1147 (47.9)		
Underweight based on body mass index, *n* (column %)	1418 (43.0)	198 (44.7)	281 (42.2)	939 (43.0)	0.410	0.500
Missing height and/or weight, *n*	268	22	36	210		
Prior AIDS diagnosis, *n* (column %)	1548 (43.5)	213 (45.8)	310 (44.2)	1025 (42.8)	0.580	0.228
Initial NRTI backbone, *n* (column %)						
Tenofovir + lamivudine/emtricitabine	3319 (93.4)	435 (94.2)	656 (93.5)	2228 (93.3)	0.339	0.559
Zidovudine+ lamivudine/emtricitabine	151 (4.3)	15 (3.3)	33 (4.7)	103 (4.3)		
Other	82 (2.3)	12 (2.6)	13 (1.9)	57 (2.4)		
Missing, *n*	11	3	0	8		
Time of ART initiation, *n* (column %)						
On date of tuberculosis diagnosis or within 2 months after^b^	1503 (42.2)	177 (38.1)	238 (33.9)	1088 (45.4)	0.146	0.004
≤6 months before tuberculosis diagnosis	869 (24.4)	134 (28.8)	193 (27.5)	542 (22.6)		
>6 months before tuberculosis diagnosis	1191 (33.4)	154 (33.1)	271 (38.6)	766 (32.0)		
Recent CD4 test, *n* (column %)	1381 (38.8)	157 (33.8)	236 (33.6)	988 (41.2)	0.959	0.003
CD4 count in cells/mm^3^ among those with a CD4 test, median (IQR)	135 (50‐309)	107 (33‐214)	129 (39‐312)	142 (55‐316)	0.166	0.003
Recent viral load test among those on ART >6 months, *n* (column %)	740 (62.1)	124 (80.5)	161 (59.4)	455 (59.4)	<0.001	<0.001
Viral load among those on ART >6 months with a VL test, *n* (column %)						
≥1000 copies/ml	178 (24.1)	28 (22.6)	38 (23.6)	112 (24.6)	0.839	0.639
<1000 copies/ml	562 (76.0)	96 (77.4)	123 (76.4)	343 (75.4)		
Site of tuberculosis disease, *n* (column %)						
Pulmonary only	2164 (60.7)	336 (72.3)	456 (65.0)	1372 (57.3)	<0.001	<0.001
Extrapulmonary	278 (7.8)	13 (2.8)	60 (8.6)	205 (8.6)		
Unknown (proxy diagnosis)	1121 (31.5)	116 (25.0)	186 (26.5)	819 (34.2)		
Documentation of initiating HRZE regimen, *n* (column %)^c^	2250 (71.8)	270 (61.0)	513 (75.1)	1467 (73.1)	<0.001	<0.001
*Viral load test availability in 12 months after tuberculosis diagnosis*						
Viral load test among entire sample, *n* (column %)	2079 (58.4)	302 (65.0)	438 (62.4)	1339 (55.9)	0.375	<0.001
Number of viral load tests, *n* (column %)						
1	1580 (76.0)	245 (81.1)	313 (71.5)	1022 (76.3)	0.003	0.072
≥2	499 (24.0)	57 (18.9)	125 (28.5)	317 (23.7)		
Viral load test among patients whose earliest event was not loss to program or death, *n* (column %)	2041 (79.7)^d^	301 (84.6)	425 (84.8)	1315 (77.1)	0.911	0.002

Abbreviations: ART, antiretroviral therapy; C‐EFV, contemporaneous efavirenz; DTG, dolutegravir; H‐EFV, historical efavirenz; HRZE, isoniazid, rifampicin, pyrazinamide and ethambutol; IQR, interquartile range; NRTI, nucleoside/nucleotide reverse transcriptase inhibitor.

^a^ Complete case analysis using only available data was used to generate *p*‐values.

^b^ Median (IQR) days from tuberculosis diagnosis to ART initiation: 5 (0–17).

^c^ Limited to sites reporting ≥1 patient documented to receive HRZE (*n* = 3134).

^d^ Because loss to program or death may preclude viral load testing, this sample (*n* = 2562) was limited to those whose earliest event during follow‐up was not loss to program or death, that is earliest event was viral suppression or switching ART regimen, or had no event. The numerator excludes the 38 patients who had a viral load test without suppression before documented loss to program or death.

Patients were categorized into the following exposure groups: DTG (*n* = 465), C‐EFV (*n* = 702) and H‐EFV (*n* = 2396). Overall, 93% were receiving an initial NRTI backbone of tenofovir plus lamivudine or emtricitabine, with no significant differences between DTG and EFV groups. Most patients in the DTG group were at sites that reported using twice‐daily dolutegravir dosing with rifampicin (432 [93%]), eight (2%) were at sites reporting only once‐daily dosing and 25 (5%) were at sites that did not participate in the survey.

When comparing patient characteristics of DTG with both EFV groups, there were significant differences in age group, sex and site of tuberculosis disease, and when comparing DTG with the H‐EFV group, there were also significant differences in timing of ART initiation and CD4 count (Table 1). Compared with both EFV groups, patients in the DTG group were more often in older age groups (e.g. 26% vs. 16% ≥50 years), male (67% vs. 46–52%) and had documentation of pulmonary only tuberculosis (72% vs. 57–65%). Compared with H‐EFV, patients in the DTG group less often initiated ART at the time of tuberculosis diagnosis (38% vs. 45%) and more often did so within 6 months prior (29% vs. 23%). CD4 counts were higher in the H‐EFV group (median 142 cells/mm^3^) versus DTG group (median 107 cells/mm^3^); however, patients in the DTG group less often had a CD4 test (34% vs. 41%). Although ART‐experienced patients in the DTG group were more likely than EFV groups to have a baseline VL test (81% vs. 59%), there were no significant differences in viral suppression. Documentation of HRZE was significantly lower in the DTG group (61%) compared with both EFV groups (73–75%).

**Figure 3:**
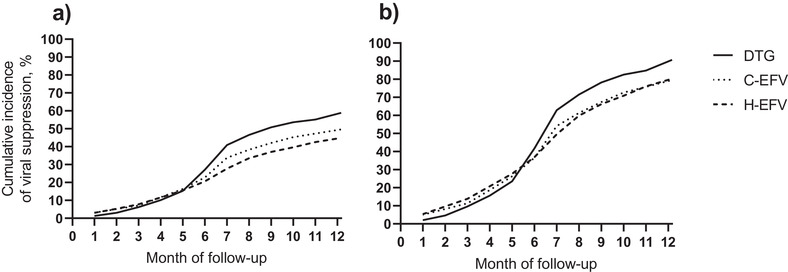
Crude cumulative incidence of viral suppression in the 12 months after tuberculosis diagnosis stratified by exposure group among (a) the entire sample and (b) patients with viral load ascertained in the 12 months after tuberculosis diagnosis. Abbreviations: C‐EFV, contemporaneous efavirenz; DTG, dolutegravir; H‐EFV, historical efavirenz. The horizontal axes represent month of follow‐up after tuberculosis diagnosis through 12 months. The vertical axes represent the crude cumulative incidence proportion of patients with viral suppression (viral load <1000 copies/ml) at each month during follow‐up. The left panel (a) includes the entire sample (*n* = 3563) regardless of viral load testing and the right panel (b) includes the subgroup of patients who had viral load ascertained in the 12 months after their tuberculosis diagnosis (*n* = 2079). In the graph, the solid lines represent the DTG group, the dotted lines represent the C‐EFV group and the dashed lines represent the H‐EFV group. Median (interquartile range) months until viral suppression among those with the outcome was 6.1 (4.9–7.5) for DTG, 6.1 (4.2–7.6) for C‐EFV and 6.1 (3.9–8.0) for H‐EFV.

At the end of 12‐month follow‐up, the cumulative incidence of viral suppression was 58.9% for DTG, 49.6% for C‐EFV and 44.9% for H‐EFV; the cumulative incidence of switching ART regimen was 4.1% for DTG, 10.0% for C‐EFV and 5.7% for H‐EFV; and the cumulative incidence of loss to program or death was 23.4% for DTG, 28.6% for C‐EFV and 28.8% for H‐EFV (Table 2). When restricting to the 2079 patients who had VL ascertained in the 12 months after tuberculosis diagnosis, the cumulative incidence of viral suppression was 90.7% for DTG, 79.5% for C‐EFV and 80.3% for H‐EFV. Median time until viral suppression was about 6 months for all groups and differences in viral suppression between DTG and EFV groups emerged just before this time (Figure 3). Cumulative incidence of viral suppression by exposure group was similar in magnitude among subgroups (Table 3). In multivariable regression, DTG was associated with significantly greater hazards of viral suppression compared with the C‐EFV (adjusted subdistribution hazard ratio [aSHR] 1.28, 95% CI: 1.08–1.51) and H‐EFV (aSHR 1.46, 95% CI: 1.28–1.67) groups (Table 4). Hazard ratios had the same direction and were similar in magnitude when limited to patients with VL ascertained and by subgroup, with some differences in statistical significance.

**Table 2:** Crude cumulative incidence of viral suppression and competing events during 12‐month follow‐up for dolutegravir and efavirenz exposure groups

	Entire sample (*n* = 3563)			Patients with viral load ascertained in 12 months after tuberculosis diagnosis (*n* = 2079)		
Outcome	DTG (*n* = 465)	C‐EFV (*n* = 702)	H‐EFV (*n* = 2396)	DTG (*n* = 302)	C‐EFV (*n* = 438)	H‐EFV (*n* = 1339)
Viral suppression						
Cumulative incidence, % (95% CI)	58.9 (54.3–63.3)	49.6 (45.8–53.2)	44.9 (42.9–46.9)	90.7 (86.8–93.5)	79.5 (75.3–83.0)	80.3 (78.1–82.3)
Outcome, *n*	274	348	1075	274	348	1075
Competing events, *n*	128	271	828	17^a^	65^a^	125^a^
Censored, *n*	63	83	493	11^b^	25^b^	139^b^
Switch ART regimen						
Cumulative incidence, % (95% CI)	4.1 (2.6–6.2)	10.0 (7.9–12.3)	5.7 (4.8–6.7)	5.3 (3.2–8.2)	11.9 (9.1–15.1)	7.5 (6.2–9.0)
Outcome, *n*	19	70	137	16	52	101
Other events, *n*	383	549	1766	275	361	1099
Censored, *n*	63	83	493	11	25	139
Loss to program or death						
Cumulative incidence, % (95% CI)	23.4 (19.7–27.4)	28.6 (25.3–32.0)	28.8 (27.0–30.7)	0.3 (0.03–1.8)	3.0 (1.7–4.9)	1.8 (1.2–2.6)
Outcome, *n*	109	201	691	1	13	24
Other events, *n*	293	418	1212	290	400	1176
Censored, *n*	63	83	493	11	25	139

Abbreviations: ART, antiretroviral therapy; C‐EFV, contemporaneous efavirenz; CI, confidence interval; DTG, dolutegravir; H‐EFV, historical efavirenz.

^a^ Patients who had a viral load test without suppression and then were lost to program or died, and patients who switched ART regimen and had a viral load test without suppression before switching or had a viral load test with or without suppression after switching.

^b^ Patients who had a viral load test without suppression and no competing event.

**Table 3:** Crude cumulative incidence of viral suppression by subgroup during 12‐month follow‐up for dolutegravir and efavirenz exposure groups

	Entire sample			Patients with viral load ascertained in 12 months after tuberculosis diagnosis		
Subgroup	DTG	C‐EFV	H‐EFV	DTG	C‐EFV	H‐EFV
Newly initiated ART	*n* = 177	*n* = 238	*n* = 1088	*n* = 105	*n* = 146	*n* = 517
Cumulative incidence, % (95% CI)	53.1 (45.4–60.2)	49.6 (43.1–55.8)	39.8 (36.9–42.7)	89.5 (81.6–94.2)	80.8 (73.3–86.4)	83.8 (80.3–86.7)
Outcome, *n*	94	118	433	94	118	433
Competing events, *n*	66	93	413	7	20	36
Censored, *n*	17	27	242	4	8	48
Initiated ART ≤6 months before tuberculosis diagnosis	*n* = 134	*n* = 193	*n* = 542	*n* = 86	*n* = 118	*n* = 330
Cumulative incidence, % (95% CI)	61.2 (52.3–68.9)	50.3 (43.0–57.1)	50.0 (45.7–54.1)	95.4 (87.2–98.4)	82.2 (73.8–88.1)	82.1 (77.5–85.9)
Outcome, *n*	82	97	271	82	97	271
Competing events, *n*	33	78	170	2	15	21
Censored, *n*	19	18	101	2	6	38
Initiated ART >6 months before tuberculosis diagnosis^a^	*n* = 154	*n* = 271	*n* = 766	*n* = 111	*n* = 174	*n* = 492
Cumulative incidence, % (95% CI)	63.6 (55.4–70.7)	49.1 (43.0–54.9)	48.4 (44.9–51.9)	88.3 (80.5–93.1)	76.4 (69.3–82.1)	75.4 (71.3–79.0)
Outcome, *n*	98	133	371	98	133	371
Competing events, *n*	29	100	245	8	30	68
Censored, *n*	27	38	150	5	11	53
HRZE documented	*n* = 270	*n* = 513	*n* = 1467	*n* = 188	*n* = 325	*n* = 903
Cumulative incidence, % (95% CI)	61.5 (55.4–67.0)	50.7 (46.3–54.9)	49.4 (46.8–51.9)	88.3 (82.7–92.2)	80.0 (75.2–84.0)	80.2 (77.4–82.6)
Outcome, *n*	166	260	724	166	260	724
Competing events, *n*	71	189	503	15	43	87
Censored, *n*	33	64	240	7	22	92
HRZE not documented	*n* = 173	*n* = 170	*n* = 541	*n* = 106	*n* = 102	*n* = 294
Cumulative incidence, % (95% CI)	57.8 (50.0–64.8)	45.9 (38.2–53.2)	42.3 (38.1–46.5)	94.3 (87.4–97.5)	76.5 (66.7–83.7)	77.9 (72.7–82.2)
Outcome, *n*	100	78	229	100	78	229
Competing events, *n*	52	76	208	2	21	32
Censored, *n*	21	16	104	4	3	33
Pulmonary only tuberculosis	*n* = 336	*n* = 456	*n* = 1372	*n* = 219	*n* = 296	*n* = 742
Cumulative incidence, % (95% CI)	59.2 (53.8–64.3)	50.4 (45.8–54.9)	44.0 (41.4–46.6)	90.9 (86.1–94.1)	77.7 (72.5–82.1)	81.4 (78.4–84.0)
Outcome, *n*	199	230	604	199	230	604
Competing events, *n*	92	169	463	10	49	67
Censored, *n*	45	57	305	10	17	71
Extrapulmonary tuberculosis	*n* = 13	*n* = 60	*n* = 205	*n* = 8	*n* = 38	*n* = 117
Cumulative incidence, % (95% CI)	53.9 (22.2–77.5)	50.0 (36.5–62.1)	47.3 (40.3–54.0)	^b^	79.0 (61.1–89.3)	82.9 (74.6–88.7)
Outcome, *n*	7	30	97	7	30	97
Competing events, *n*	4	23	71	1	5	8
Censored, *n*	2	7	37	0	3	12
Proxy diagnosis	*n* = 116	*n* = 186	*n* = 819	*n* = 75	*n* = 104	*n* = 480
Cumulative incidence, % (95% CI)	58.6 (49.0–67.0)	47.3 (39.9–54.3)	45.7 (42.2–49.0)	90.7 (80.7–95.6)	84.6 (75.9–90.4)	77.9 (73.9–81.4)
Outcome, *n*	68	88	374	68	88	374
Competing events, *n*	32	79	294	6	11	50
Censored, *n*	16	19	151	1	5	56

Abbreviations: ART, antiretroviral therapy; C‐EFV, contemporaneous efavirenz; CI confidence interval; DTG, dolutegravir; H‐EFV, historical efavirenz; HRZE, isoniazid, rifampicin, pyrazinamide and ethambutol.

^a^ Analyses stratified by recent viral load among those initiating ART >6 months before tuberculosis diagnosis are presented in Table S6.

^b^ Unable to estimate.

**Table 4:** Hazards regression models for the associations between dolutegravir and efavirenz groups with viral suppression during 12‐month follow‐up

	Entire sample				Patients with viral load ascertained in 12 months after tuberculosis diagnosis			
Sample and comparison	*n*	aSHR	95% CI	*p*‐value	*n*	aSHR	95% CI	*p*‐value
Overall								
DTG versus C‐EFV^a^	1167	1.28	1.08–1.51	0.004	740	1.31	1.11–1.54	0.002
DTG versus H‐EFV^b^	2861	1.47	1.28–1.68	<0.001	1641	1.31	1.15–1.49	<0.001
Newly initiated ART								
DTG versus C‐EFV^a^	415	1.19	0.89–1.59	0.246	251	1.43	1.06–1.94	0.019
DTG versus H‐EFV^b^	1265	1.50	1.20–1.89	<0.001	622	1.24	0.99–1.56	0.063
Initiated ART ≤6 months before tuberculosis diagnosis								
DTG versus C‐EFV^a^	327	1.29	0.95–1.75	0.107	204	1.32	0.98–1.79	0.072
DTG versus H‐EFV^b^	676	1.27	0.98–1.63	0.068	416	1.25	0.99–1.56	0.058
Initiated ART >6 months before tuberculosis diagnosis^c^								
DTG versus C‐EFV^a^	425	1.36	1.03–1.78	0.029	285	1.16	0.89–1.51	0.271
DTG versus H‐EFV^b^	920	1.54	1.23–1.93	<0.001	603	1.40	1.13–1.74	0.002
HRZE documented								
DTG versus C‐EFV^a^	783	1.39	1.13–1.70	0.002	513	1.28	1.05–1.57	0.017
DTG versus H‐EFV^b^	1737	1.35	1.14–1.61	<0.001	1091	1.28	1.08–1.52	0.004
HRZE not documented								
DTG versus C‐EFV^a^	343	1.24	0.90–1.71	0.181	208	1.32	0.94–1.86	0.105
DTG versus H‐EFV^b^	714	1.53	1.20–1.94	<0.001	400	1.58	1.26–1.97	<0.001
Pulmonary only tuberculosis								
DTG versus C‐EFV^a^	792	1.24	1.02–1.51	0.035	515	1.33	1.09–1.61	0.004
DTG versus H‐EFV^b^	1708	1.46	1.25–1.71	<0.001	961	1.24	1.06–1.43	0.006
Extrapulmonary tuberculosis								
DTG versus C‐EFV^a^	73	1.27	0.55–2.93	0.571	46	1.53	0.66–3.56	0.319
DTG versus H‐EFV^b^	218	1.17	0.53–2.46	0.733	125	1.07	0.49–2.35	0.869
Proxy diagnosis								
DTG versus C‐EFV^a^	302	1.43	1.02–2.00	0.036	179	1.31	0.94–1.83	0.114
DTG versus H‐EFV^b^	935	1.54	1.18–2.02	0.002	555	1.68	1.29–2.19	<0.001

Abbreviations: ART, antiretroviral therapy; aSHR, adjusted subdistribution hazard ratio; C‐EFV, contemporaneous efavirenz; CI confidence interval; DTG, dolutegravir; H‐EFV, historical efavirenz; HRZE, isoniazid, rifampicin, pyrazinamide and ethambutol.

^a^ Models adjusted for age group, sex and tuberculosis disease site (if not stratified by this variable).

^b^ Models adjusted for age group, sex, time of ART initiation (if not stratified by this variable) and tuberculosis disease site (if not stratified by this variable).

^c^ Analyses stratified by recent viral load among those initiating ART >6 months before tuberculosis diagnosis are presented in Table S7.

Results were similar with the main analysis when redefining viral suppression as <400 copies/ml (Tables S2 and S3) and when stratifying by the number of VL tests in the 12 months after tuberculosis diagnosis (Tables S4 and S5). When stratifying by most recent baseline VL among patients initiating ART >6 before tuberculosis diagnosis (Tables S6 and S7), differences between DTG and EFV groups appeared to be driven by those with a VL ≥1000 copies/ml or no VL test. Viral suppression was similar by exposure group for those with a recent baseline VL <1000 copies/ml.

## 4 DISCUSSION

In a broad sample of HIV clinics in sub‐Saharan Africa, >95% of sites reported prescribing dolutegravir‐containing regimens to PWH receiving rifampicin as part of tuberculosis treatment and 90% of these sites reported using twice‐daily dosing of dolutegravir to counteract the drug–drug interaction. Dolutegravir is being widely rolled out as a once‐daily fixed dose combination tablet with tenofovir disoproxil fumarate and lamivudine (“TLD”) [3]. Therefore, national programs need to acquire 50 mg tablets and dispensaries need to stock them to accommodate twice‐daily dosing. Among sites using twice‐daily dosing, >95% reported having dolutegravir 50 mg tablets available.

In the cohort study, a large proportion (91%) of patients receiving dolutegravir who had VL ascertained in the 12 months after tuberculosis diagnosis had viral suppression, which was lower (59%) for the entire sample that fully considered loss to program or death, which could preclude VL testing, and patients who did not have VL testing for other reasons. For both samples, dolutegravir was significantly associated with improved viral suppression compared with efavirenz. When stratifying by subgroup, there were some differences in statistical significance for comparisons, but overall, results were similar in magnitude. In the INSPIRING trial, 48‐week viral suppression (<50 copies/ml) was similar in magnitude for both the dolutegravir (75%) and efavirenz (82%) arms among participants newly initiating ART; however, this trial was not powered to statistically compare viral suppression between treatment arms [16]. Real‐world evidence from Botswana [27] and France [28] supports similar virologic effectiveness of dolutegravir compared with efavirenz among patients with tuberculosis co‐infection receiving rifampicin.

The finding that viral suppression with dolutegravir was at least as good as (and possibly better) compared with efavirenz is supportive of HIV treatment programs already routinely prescribing dolutegravir to patients with tuberculosis co‐infection. In some settings, efavirenz remains preferred for patients initiating ART at the time of tuberculosis diagnosis to avoid the drug–drug interaction with rifampicin [29] and our results support that it remains a reasonable alternative. Our study provides reassurance about the virologic effectiveness of dolutegravir in the context of this drug–drug interaction and supports further expanding access to PWH in accordance with international guidelines, including those initiating rifampicin as part of tuberculosis treatment [1, 2]. Despite these positive findings, the high proportion of patients who were lost to program or died, regardless of ART regimen, emphasizes the need to further improve outcomes among PWH who have tuberculosis co‐infection.

The results from our cohort study should be interpreted in the context of some limitations. We attempted to adjust for potential confounders in regression analyses, which we considered to be patient characteristics that were significantly different between exposure groups. Nevertheless, because of the observational study design, unobserved confounders may be present. The comparison of DTG with H‐EFV allowed for potentially less selection bias than the comparison with C‐EFV, as dolutegravir was not yet available when these patients had a tuberculosis diagnosis. However, follow‐up in the H‐EFV group began as early as 2015 and thus, the lower viral suppression in this group may have been related to changes in VL testing availability and practices over time, including the use of targeted rather than routine VL monitoring [30]. Despite this, associations were generally similar between DTG and both C‐EFV and H‐EFV groups.

As we leveraged routine clinical data, misclassification of some variables and missing data related to erroneous or incomplete documentation is anticipated. For the inclusion criteria, it may be possible that a tuberculosis diagnosis was incorrectly documented. It may also be possible that some patients had drug resistant tuberculosis and were not receiving rifampicin; however, such treatment is often provided off‐site in our setting [31]. To examine these potential sources of bias, we conducted subgroup analyses limited to those with documentation of HRZE and found that results were consistent with the main analysis.

We did not have information on dosing frequency of antiretroviral drugs, so we could not compare outcomes by dolutegravir dosing. Few patients in the DTG group were at sites that reported not using twice‐daily dosing, precluding a robust subgroup analysis. The necessity of dolutegravir 50 mg twice daily for all patients receiving concomitant rifampicin remains unresolved [27, 32, 33]; however, further evidence is anticipated from an ongoing randomized trial [34]. Genotypic resistance testing would have allowed an assessment of resistance among patients with viral non‐suppression on dolutegravir, as emergent dolutegravir resistance in the context of the drug–drug interaction with rifampicin has been described [35, 36]. We also did not have data about safety or tolerability, precluding an assessment of risks versus benefits.

## 5 CONCLUSIONS

We documented the widespread programmatic implementation of dolutegravir for patients with tuberculosis co‐infection at HIV clinics in sub‐Saharan Africa and use of the recommended twice‐daily dosing to counteract the drug–drug interaction with rifampicin. Despite this more complex regimen, dolutegravir did not negatively impact viral suppression.

## COMPETING INTERESTS

The authors declare that they have no competing interests.

## AUTHORS’ CONTRIBUTIONS

MLR and DN conceived the study. MLR, EB, MY, LF, KKW‐K, NSS and DN contributed to the study design. EB, DM‐N, RDW, CS‐W, CC, WRM, GM, CK, TT, JAM, PL, AD, CT, IR, OCE and LD contributed to data collection. MLR analysed the data and wrote the manuscript. All authors participated in the interpretation of the results, revision of the manuscript, and have read and approved the final manuscript.

## FUNDING

The International Epidemiology Databases to Evaluate AIDS (IeDEA) is supported by the U.S. National Institutes of Health's National Institute of Allergy and Infectious Diseases, the *Eunice Kennedy Shriver* National Institute of Child Health and Human Development, the National Cancer Institute, the National Institute of Mental Health, the National Institute on Drug Abuse, the National Heart, Lung, and Blood Institute, the National Institute on Alcohol Abuse and Alcoholism, the National Institute of Diabetes and Digestive and Kidney Diseases, the Fogarty International Center and the National Library of Medicine: Central Africa, U01AI096299; East Africa, U01AI069911; Southern Africa, U01AI069924; West Africa, U01AI069919. Informatics resources are supported by the Harmonist project, R24AI24872.

## DISCLOSURE

This work is solely the responsibility of the authors and does not necessarily represent the official views of the U.S. National Institutes of Health.

## Supporting information


**Appendix S1**. Members of the contributing IeDEA regions.

**Appendix S2**. Site survey of dolutegravir use among patients with tuberculosis co‐infection.

**Supplemental Table 1**. Characteristics of sites included in survey of dolutegravir use in tuberculosis co‐infection.
**Supplemental Table 2**. Cumulative incidence of viral suppression redefining viral suppression from <1000 copies/mL to <400 copies/mL (sensitivity analysis).
**Supplemental Table 3**. Hazards regression models for the associations between dolutegravir and efavirenz groups with viral suppression redefined from <1000 copies/mL to <400 copies/mL (sensitivity analysis).
**Supplemental Table 4**. Cumulative incidence of viral suppression stratified by number of viral load tests in the 12 months after tuberculosis diagnosis (sensitivity analysis).
**Supplemental Table 5**. Hazards regression models for the associations between dolutegravir and efavirenz groups stratified by number of viral load tests in the 12 months after tuberculosis diagnosis (sensitivity analysis).
**Supplemental Table 6**. Cumulative incidence of viral suppression stratified by baseline recent viral load among patients initiating antiretroviral therapy >6 months before tuberculosis diagnosis (sensitivity analysis).
**Supplemental Table 7**. Hazards regression models for the associations between dolutegravir and efavirenz groups stratified by baseline recent viral load among patients initiating antiretroviral therapy >6 months before tuberculosis diagnosis (sensitivity analysis).

## Data Availability

The data that support the findings of this study are available from the corresponding author upon reasonable request. Individuals who wish to request access to data from the IeDEA consortium for research purposes may submit a concept proposal, which is detailed at https://www.iedea.org/.
