# Supplementary material for: Real‐world use and outcomes of dolutegravir‐containing antiretroviral therapy in HIV and tuberculosis co‐infection: a site survey and cohort study in sub‐Saharan Africa
Source: J Int AIDS Soc. 2022 Jul 18;25(7):e25961. doi: 10.1002/jia2.25961 (PMC9289708; doi:10.1002/jia2.25961)
Supplement: Supplementary file 2 — Appendix S2. Site survey of dolutegravir use among patients with tuberculosis co‐infection. [file JIA2-25-e25961-s003.docx]

**Appendix 2. Site survey of dolutegravir use among patients with tuberculosis co-infection**

We are conducting a survey of sites in IeDEA to understand how dolutegravir is used among patients with HIV and tuberculosis (TB) co-infection. We would sincerely appreciate your participation.

To answer the questions in this survey, please consult clinicians, pharmacy staff, and others as needed. Note that all questions are specific to the management of drug-susceptible TB in adults and children weighing at least 20 kg. Responses should reflect current practices at your site.

| **QUESTIONS** | | **RESPONSES** | | |
| --- | --- | --- | --- | --- |
| **1. Respondent information and survey screening** | | | | |
| 1.1 Name of your site | |  | | |
| 1.2 Name of person completing this survey | |  | | |
| 1.3 Email address of the person completing the survey | |  | | |
| 1.4 Please enter the date this survey was completed | | / / (DD / MM / YYYY) | | |
| 1.5 What is your title? | | ☐ Head Clinician/Clinical Officer In-Charge  ☐ Other clinician  ☐ Site Manager  ☐ Site Data Manager  ☐ Head Nurse  ☐ Other (specify) ___________________________ | | |
| 1.6 Is dolutegravir used at your site? | | ☐ Yes  ☐ No (END SURVEY)  ☐ Do not know (END SURVEY) | | |
| 1.7 Does your site provide antiretroviral therapy (ART) to patients with HIV/TB co-infection? | | ☐ Yes  ☐ No (END SURVEY)  ☐ Do not know (END SURVEY) | | |
| **2. Dolutegravir availability and use among patients with TB co-infection** | | | | |
| 2.1 Are dolutegravir 50 mg tablets currently available at your site?  *Note, these are tablets that contain ONLY dolutegravir and no other medication.* | | | ☐ Yes  ☐ No  ☐ Do not know | |
| 2.2 At your site, is dolutegravir-containing ART ever prescribed to patients who are also receiving rifampicin for tuberculosis treatment? | | | ☐ Yes  ☐ No (SKIP TO Q4.1)  ☐ Do not know | |
| **3. Concurrent dolutegravir and rifampicin use among patients with TB co-infection** | | | | |
| 3.1 How does your site manage patients with HIV/TB co-infection receiving concomitant rifampicin and dolutegravir?  *Check one best response* | ☐ All patients receive a once-daily dolutegravir-containing regimen (e.g., TLD) (END SURVEY)  ☐ All patients start a second daily dose of dolutegravir 50 mg during while receiving rifampicin (END SURVEY)  ☐ Some patients receive a once-daily dolutegravir-containing regimen and some start a second daily dose of dolutegravir 50 mg | | | |
| 3.1a Which groups of patients with HIV/TB co-infection do not routinely receive an increased dolutegravir dose (i.e., twice daily) during rifampicin-containing TB treatment?  *Check all that apply.* | ☐ Patients with low body weight (END SURVEY)  ☐ Patients at risk for suboptimal medication adherence to ART (END SURVEY)  ☐ Patients at risk for suboptimal medication adherence to TB treatment (END SURVEY)  ☐ Patients with a history of adverse events or tolerability issues related to ART (END SURVEY)  ☐ Other (specify) ___________________________ (END SURVEY) | | | |
| **4. Management of ART among patients with TB co-infection** | | | | |
| 4.1 Are patients with HIV/TB co-infection who have not yet started ART at the time of TB diagnosis routinely initiated on a non-dolutegravir-containing regimen (e.g., efavirenz-tenofovir-lamivudine) while on rifampicin-containing TB treatment? | | | | ☐ Yes  ☐ No  ☐ Do not know |
| 4.2 Are patients with HIV/TB co-infection who are already on a dolutegravir-containing regimen at the time of TB diagnosis routinely switched to a non-dolutegravir-containing regimen (e.g., efavirenz-tenofovir-lamivudine) while on rifampicin-containing TB treatment? | | | | ☐ Yes  ☐ No  ☐ Do not know |
| 4.3 Are patients with HIV/TB co-infection who are already on a dolutegravir-containing regimen at the time of TB diagnosis routinely initiated on TB treatment that contains an alternative rifamycin (e.g., rifabutin)? | | | | ☐ Yes  ☐ No  ☐ Do not know  ☐ Not applicable (site does not initiate TB medications) |
