# Supplementary material for: Real‐world use and outcomes of dolutegravir‐containing antiretroviral therapy in HIV and tuberculosis co‐infection: a site survey and cohort study in sub‐Saharan Africa
Source: J Int AIDS Soc. 2022 Jul 18;25(7):e25961. doi: 10.1002/jia2.25961 (PMC9289708; doi:10.1002/jia2.25961)
Supplement: Supplementary file 3 — Supplemental Table 1. Characteristics of sites included in survey of dolutegravir use in tuberculosis co‐infection. Supplemental Table 2. Cumulative incidence of viral suppression redefining viral suppression from <1000 copies/mL to <400 copies/mL (sensitivity analysis). Supplemental Table 3. Hazards regression models for the associations between dolutegravir and efavirenz groups with viral suppression redefined from <1000 copies/mL to <400 copies/mL (sensitivity analysis). Supplemental Table 4. Cumulative incidence of viral suppression stratified by number of viral load tests in the 12 months after tuberculosis diagnosis (sensitivity analysis). Supplemental Table 5. Hazards regression models for the associations between dolutegravir and efavirenz groups stratified by number of viral load tests in the 12 months after tuberculosis diagnosis (sensitivity analysis). Supplemental Table 6. Cumulative incidence of viral suppression stratified by baseline recent viral load among patients initiating antiretroviral therapy >6 months before tuberculosis diagnosis (sensitivity analysis). Supplemental Table 7. Hazards regression models for the associations between dolutegravir and efavirenz groups stratified by baseline recent viral load among patients initiating antiretroviral therapy >6 months before tuberculosis diagnosis (sensitivity analysis). [file JIA2-25-e25961-s001.docx]

**Supplemental Table 1. Characteristics of sites included in survey of dolutegravir use in tuberculosis co-infection**

|  | **n (%)** |
| --- | --- |
| **Total** | 90 (100%) |
|  |  |
| **IeDEA region and country** |  |
| **Central Africa** | 20 (22%) |
| Burundi | 3 |
| Cameroon | 3 |
| Republic of the Congo | 2 |
| Democratic Republic of the Congo | 1 |
| Rwanda | 11 |
| **East Africa** | 37 (41%) |
| Kenya | 34 |
| Uganda | 3 |
| **Southern Africa** | 22 (24%) |
| Malawi | 2 |
| South Africa | 10 |
| Zambia | 5 |
| Zimbabwe | 5 |
| **West Africa** | 11 (12%) |
| Benin | 1 |
| Burkina Faso | 1 |
| Côte d’Ivoire | 5 |
| Ghana | 1 |
| Nigeria | 1 |
| Senegal | 1 |
| Togo | 1 |
| **Urbanicity** |  |
| Urban | 61 (69%) |
| Rural | 27 (31%) |
| Unknown | 2 |
| **Patient population** |  |
| Adults only | 11 (12%) |
| Children only | 9 (10%) |
| Both adults and children | 69 (78%) |
| Unknown | 1 |
| **Level of care** |  |
| Primary | 47 (55%) |
| Secondary | 13 (15%) |
| Tertiary | 25 (29%) |
| Unknown | 5 |

**Supplemental table 2. Cumulative incidence of viral suppression redefining viral suppression from <1000 copies/mL to <400 copies/mL (sensitivity analysis)**

|  | **Entire sample (n=3563)** | | | **Patients with viral load ascertained in 12 months after tuberculosis diagnosis (n=2079)** | | |
| --- | --- | --- | --- | --- | --- | --- |
|  | **DTG (n=465)** | **C-EFV (n=702)** | **H-EFV (n=2396)** | **DTG (n=302)** | **C-EFV (n=438)** | **H-EFV (n=1339)** |
| Cumulative incidence, % (95% CI) | 57.9 (53.2-62.2) | 47.3 (43.6-50.9) | 42.3 (40.3-44.3) | 89.1 (84.9-92.1) | 75.8 (71.5-79.6) | 75.7 (73.3-77.9) |
| Outcome, n | 269 | 332 | 1013 | 269 | 332 | 1013 |
| Competing events, n | 130 | 279 | 832 | 19 | 73 | 129 |
| Censored, n | 66 | 91 | 551 | 14 | 33 | 197 |

C-EFV, contemporaneous efavirenz; CI confidence interval; DTG, dolutegravir; H-EFV, historical efavirenz.

**Supplemental table 3. Hazards regression models for the associations between dolutegravir and efavirenz groups with viral suppression redefined from <1000 copies/mL to <400 copies/mL (sensitivity analysis)**

|  | **Entire sample (n=3563)** | | | | **Patients with viral load ascertained in 12 months after tuberculosis diagnosis (n=2079)** | | | |
| --- | --- | --- | --- | --- | --- | --- | --- | --- |
| **Comparison** | **n** | **aSHR** | **95% CI** | **p-value** | **n** | **aSHR** | **95% CI** | **p-value** |
| DTG vs. C-EFV^†^ | 1167 | 1.33 | 1.12-1.57 | 0.001 | 740 | 1.37 | 1.16-1.62 | <0.001 |
| DTG vs. H-EFV^‡^ | 2861 | 1.52 | 1.33-1.74 | <0.001 | 1641 | 1.35 | 1.19-1.54 | <0.001 |

adjusted subdistribution hazard ratio; C-EFV, contemporaneous efavirenz; CI confidence interval; DTG, dolutegravir; H-EFV, historical efavirenz.

^†^Models adjusted for age group, sex, and tuberculosis disease site.

^‡^Models adjusted for age group, sex, time of antiretroviral therapy initiation, and tuberculosis disease site.

**Supplemental table 4. Cumulative incidence of viral suppression stratified by number of viral load tests in the 12 months after tuberculosis diagnosis (sensitivity analysis)**

|  | **1 Viral load test  (n=1580)** | | | **≥2 Viral load tests  (n=499)** | | |
| --- | --- | --- | --- | --- | --- | --- |
|  | **DTG (n=245)** | **C-EFV (n=313)** | **H-EFV (n=1022)** | **DTG (n=57)** | **C-EFV (n=125)** | **H-EFV (n=317)** |
| Cumulative incidence, % (95% CI) | 91.4 (87.1-94.4) | 80.2 (75.3-84.2) | 81.6 (79.1-83.9) | 87.7 (74.7-94.3) | 77.6 (69.0-84.1) | 76.0 (70.9-80.4) |
| Outcome, n | 224 | 251 | 834 | 50 | 97 | 241 |
| Competing events, n | 11 | 41 | 91 | 6 | 24 | 34 |
| Censored, n | 10 | 21 | 97 | 1 | 4 | 42 |

C-EFV, contemporaneous efavirenz; CI confidence interval; DTG, dolutegravir; H-EFV, historical efavirenz.

**Supplemental table 5. Hazards regression models for the associations between dolutegravir and efavirenz groups stratified by number of viral load tests in the 12 months after tuberculosis diagnosis (sensitivity analysis)**

| **Sample and comparison** | **n** | **aSHR** | **95% CI** | **p-value** |
| --- | --- | --- | --- | --- |
| **1 Viral load test  (n=1580)** |  |  |  |  |
| DTG vs. C-EFV^†^ | 558 | 1.36 | 1.12-1.65 | 0.002 |
| DTG vs. H-EFV^‡^ | 1276 | 1.37 | 1.18-1.59 | <0.001 |
| **≥2 Viral load tests  (n=499)** |  |  |  |  |
| DTG vs. C-EFV^†^ | 182 | 1.26 | 0.90-1.77 | 0.177 |
| DTG vs. H-EFV^‡^ | 374 | 1.13 | 0.85-1.52 | 0.401 |

aSHR, adjusted subdistribution hazard ratio; C-EFV, contemporaneous efavirenz; CI confidence interval; DTG, dolutegravir; H-EFV, historical efavirenz.

^†^Models adjusted for age group, sex, and tuberculosis disease site.

^‡^Models adjusted for age group, sex, time of antiretroviral therapy initiation, and tuberculosis disease site.

**Supplemental table 6. Cumulative incidence of viral suppression stratified by baseline recent viral load among patients initiating antiretroviral therapy >6 months before tuberculosis diagnosis (sensitivity analysis)**

|  | **Entire sample (n=1191)** | | | **Patients with viral load ascertained in 12 months after tuberculosis diagnosis (n=777)** | | |
| --- | --- | --- | --- | --- | --- | --- |
|  | **DTG** | **C-EFV** | **H-EFV** | **DTG** | **C-EFV** | **H-EFV** |
| **Recent viral load before tuberculosis diagnosis <1000 copies/mL** | n=96 | n=123 | n=343 | n=73 | n=88 | n=244 |
| Cumulative incidence, % (95% CI) | 69.8 (56.4-78.0) | 65.0 (55.8-72.8) | 63.6 (58.2-68.4) | 91.8 (82.0-96.4) | 90.0 (81.7-95.6) | 89.3 (84.7-92.7) |
| Outcome, n | 67 | 80 | 218 | 67 | 80 | 218 |
| Competing events, n | 15 | 31 | 76 | 5 | 8 | 16 |
| Censored, n | 14 | 12 | 49 | 1 | 0 | 10 |
| **Recent viral load before tuberculosis diagnosis ≥1000 copies/mL** | n=28 | n=38 | n=112 | n=19 | n=26 | n=74 |
| Cumulative incidence, % (95% CI) | 46.4 (27.0-63.8) | 26.3 (13.3-41.3) | 24.1 (16.6-32.4) | 68.4 (41.0-85.1) | 38.5 (19.5-57.2) | 36.5 (25.6-47.4) |
| Outcome, n | 13 | 10 | 27 | 13 | 10 | 27 |
| Competing events, n | 7 | 23 | 51 | 2 | 13 | 24 |
| Censored, n | 8 | 5 | 34 | 4 | 3 | 23 |
| **No recent viral load test** | n=30 | n=110 | n=311 | n=19 | n=60 | n=174 |
| Cumulative incidence, % (95% CI) | 60.0 (39.6-75.5) | 39.1 (29.9-48.1) | 40.5 (35.0-45.9) | ^†^ | 71.7 (58.1-81.5) | 72.4 (65.1-78.5) |
| Outcome, n | 18 | 43 | 126 | 18 | 43 | 126 |
| Competing events, n | 7 | 46 | 118 | 1 | 9 | 28 |
| Censored, n | 5 | 21 | 67 | 0 | 8 | 20 |

C-EFV, contemporaneous efavirenz; CI confidence interval; DTG, dolutegravir; H-EFV, historical efavirenz.
^†^Unable to estimate.

**Supplemental table 7. Hazards regression models for the associations between dolutegravir and efavirenz groups stratified by baseline recent viral load among patients initiating antiretroviral therapy >6 months before tuberculosis diagnosis (sensitivity analysis)**

|  | **Entire sample (n=1191)** | | | | **Patients with viral load ascertained in 12 months after tuberculosis diagnosis (n=777)** | | | |
| --- | --- | --- | --- | --- | --- | --- | --- | --- |
| **Sample and comparison** | **n** | **aSHR** | **95% CI** | **p-value** | **n** | **aSHR** | **95% CI** | **p-value** |
| **Recent viral load before tuberculosis diagnosis <1000 copies/mL** |  |  |  |  |  |  |  |  |
| DTG vs. C-EFV^†^ | 219 | 1.10 | 0.78-1.54 | 0.587 | 161 | 0.89 | 0.64-1.25 | 0.514 |
| DTG vs. H-EFV^†^ | 439 | 1.20 | 0.90-1.60 | 0.206 | 317 | 1.13 | 0.84-1.50 | 0.424 |
| **Recent viral load before tuberculosis diagnosis ≥1000 copies/mL** |  |  |  |  |  |  |  |  |
| DTG vs. C-EFV^†^ | 66 | 2.19 | 0.92-5.21 | 0.075 | 45 | 2.11 | 0.88-5.05 | 0.093 |
| DTG vs. H-EFV^†^ | 140 | 2.40 | 1.18-4.87 | 0.015 | 93 | 2.39 | 1.22-4.68 | 0.011 |
| **No recent viral load test** |  |  |  |  |  |  |  |  |
| DTG vs. C-EFV^†^ | 140 | 1.56 | 0.90-2.69 | 0.114 | 79 | 1.56 | 0.94-2.61 | 0.088 |
| DTG vs. H-EFV^†^ | 341 | 1.77 | 1.10-2.85 | 0.020 | 193 | 1.80 | 1.19-2.72 | 0.006 |

aSHR, adjusted subdistribution hazard ratio; C-EFV, contemporaneous efavirenz; CI confidence interval; DTG, dolutegravir; H-EFV, historical efavirenz.

^†^Models adjusted for age group, sex, and tuberculosis disease site.
